# Hyperuricemia as an effect modifier of the association between metabolic phenotypes and nonalcoholic fatty liver disease in Chinese population

**DOI:** 10.1186/s12967-022-03850-5

**Published:** 2023-01-21

**Authors:** Jun Zheng, Xuerui Li, Yuan Zhang, Yuyang Miao, Qiang Zhang

**Affiliations:** 1 Department of Geriatrics, Tianjin Medical University General Hospital, Tianjin Geriatrics Institute, Anshan Road NO.154, Tianjin, 300052 China; 2 Department of Ophthalmology, Tianjin Medical University General Hospital, Tianjin, China

**Keywords:** Metabolic phenotypes, Nonalcoholic fatty liver disease, Serum uric acid levels, Population-based cross-sectional study

## Abstract

**Background:**

Different metabolic phenotypes may be related to nonalcoholic fatty liver disease (NAFLD), but such association whether modified by serum uric acid levels is unknown. We examined the association between different metabolic phenotypes and NAFLD and further explore whether hyperuricemia could modify this association.

**Methods:**

A total of 2959 participants (mean age: 55.02 years) with medical checkups were recruited from Tianjin Medical University General Hospital. Participants were categorized into four groups according to their BMI levels and metabolically healthy status: metabolically healthy normal weight (MHNW), metabolically healthy overweight or obese (MHO), metabolically unhealthy normal weight (MUNW), and metabolically unhealthy overweight or obese (MUO). Blood samples (including serum uric acid) were collected from participants after an overnight fast. NAFLD was diagnosed based on abdominal ultrasonography scanning. Data were analyzed using logistic regression models and the interaction effect model.

**Results:**

The prevalence of NAFLD in MHNW, MHO, MUNW, and MUO groups was 9.9% (7.9–12.0%), 42.8% (39.5–46.1%), 36.5% (31.2–41.9%), and 69.7% (66.8–72.6%), respectively. In multi-adjusted logistic models, the ORs (95% CIs) of NAFLD were 5.32 (4.01–7.04) for participants with MHO, 4.51 (3.17–6.40) for those with MUNW, and 13.68 (10.23–18.30) for those with MUO compared to those with MHNW. In the stratified analysis by uric acid levels, the prevalence of NAFLD was significantly higher in participants with MHO, MUNW, and MUO in the hyperuricemia group than those in the normal uric acid group, and the interaction effect of metabolic phenotypes and uric acid on NAFLD was statistical significant (*P* < 0.05).

**Conclusions:**

MHO, MUNW, and MUO were associated with higher prevalence of NAFLD. Serum uric acid levels may modify the association between metabolically phenotypes and NAFLD.

**Supplementary Information:**

The online version contains supplementary material available at 10.1186/s12967-022-03850-5.

## Background

Nonalcoholic fatty liver disease (NAFLD) affects approximately one-fifth of adults in China and 20% to 40% of the population in western countries. The global prevalence of NAFLD is increasing continually in recent years because of the increase in etiological factors [1, 2]. NAFLD encompasses a range of disease stages such as simple steatosis, nonalcoholic steatohepatitis, fibrosis, and cirrhosis, which may eventually lead to liver cancer or liver failure [3, 4]. In addition to intrahepatic injury, NAFLD is associated with an increased risk of cardiovascular events, type 2 diabetes as well as all-cause mortality [5–8]. Since NAFLD imposes a heavy economic burden on the health system, it is greatly meaningful for us to explore the predictors of NAFLD by integrating multiple traditional risk factors.

Up to now, NAFLD has been recognized as a multifactorial chronic disease that is related to hereditary, environmental, and metabolic factors [9, 10]. It is especially emphasized that excessive weight was the main culprit of the high prevalence of NAFLD [11]. A large cohort study reported that obesity itself increased the risk of NAFLD independent of metabolic status [12]. However, the metabolic characteristic of obese patients is diverse and the impact on NAFLD is imprecise. In recent years, a class of metabolic phenotypes representing the comprehensive state of the body, i.e., combining BMI level and metabolic status, has gradually emerged. For instance, the state of being overweight or obese but with normal lipid profile, glucose metabolism, blood pressure, and inflammatory markers is characterized as metabolically healthy overweight or obese (MHO phenotype) [13]. Another obesity phenotype, metabolically unhealthy overweight or obese (MUO), has been shown to be more prone to NAFLD than MHO [14]. Conversely, the MUNW phenotype (described as a state of metabolically unhealthy but normal weight) may also be associated with a high prevalence of a subset of chronic non-communicable diseases [15]. Among them, the prevalence of NAFLD varied from 5 to 80% with the number of diagnostic criteria for metabolic syndrome [16]. Given these findings, it is necessary to combine both obesity and metabolic status in assessing the risk of NAFLD.

As a common abnormal metabolic state, the incidence of hyperuricemia in China is on the rise with the continuous improvement of living standards [17]. A series of epidemiological studies revealed an association between elevated serum uric acid and occurrence or progression of NAFLD in different countries [18–20]. Conversely, the research by Takeshi Baba et al. found no significant relationship between hyperuricemia and fatty liver [21]. Besides, a meta-analysis further confirmed that hyperuricemia was not associated with the degree of fibrosis in NAFLD [22]. Meanwhile, modification of the association between hyperuricemia and NAFLD by obesity status has also been demonstrated in several studies [23]. Therefore, it is plausible that uric acid levels may play a role in the metabolic phenotypes-NAFLD association. Nevertheless, data regarding the role of hyperuricemia in metabolic phenotypes and NAFLD were relatively rare. Questions remained regarding whether and to what extent hyperuricemia may modify the association between metabolic phenotypes and NAFLD.

In the current study, we sought to examine the association between different metabolic phenotypes and NAFLD and further explore whether hyperuricemia could modify such association using data from the population-based Chinese cross-sectional study.

## Methods

### Study population

**Fig. 1:**
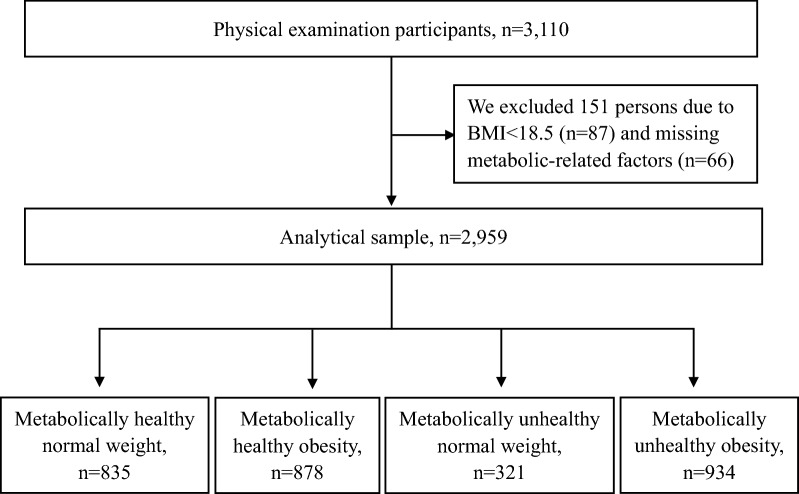
Flowchart of the study population

Study subjects were drawn from the Tianjin Medical University General Hospital who visited the Physical Examination Center for a medical checkup from January to December 2021. Among 3110 participants who completed the liver ultrasonography examination and without significant alcohol intake (more than 10 g/day for women and more than 20 g/day for men), we excluded 151 persons including 87 whose body mass index (BMI) < 18.5 kg/m^2^ and 66 with missing data on metabolic-related factors (including systolic/diastolic blood pressure [SBP/DBP], triglycerides, fasting blood glucose [FBG], low-density lipoprotein cholesterol [LDL-C], and high-density lipoprotein cholesterol [HDL-C]). Finally, 2959 participants remained for the current study (Fig. 1). Informed consent was required from all participants. The study protocol was reviewed and approved by the Ethics Committee of Tianjin Medical University General Hospital.

### Data collection

Data on anthropometric characteristics (height and weight), blood pressure, and blood samples were obtained by physical examination. A height and weight measuring device was used to measure the height and weight of the participants with light indoor clothing. Blood pressure was drawn in the sitting position after participants rested for at least 5-min using an upper arm medical electronic sphygmomanometer. Both diastolic and systolic blood pressures were measured. Blood samples (including FBG, total cholesterol, triglyceride, HDL-C, LDL-C, creatinine, alanine transaminase, total bilirubin, albumin, and uric acid) were collected in the morning after the overnight fast. All blood samples were analyzed by the enzyme method in the auto-analyzing machine and were performed by laboratory technicians in the hospital following standard laboratory procedures. Furthermore, hyperuricemia was defined as a serum uric acid level > 420 μmol/L (7.0 mg/dL) in males and > 360 μmol/L (6.0 mg/dL) in females [24].

### Assessment of metabolic phenotypes

BMI was calculated as weight in kilograms divided by height in meters squared (kg/m^2^) and was further categorized as 18.5 to 23.9 kg/m^2^, 24.0 to 27.9 kg/m^2^, and greater than or equal to 28.0 kg/m^2^ according to Chinese BMI criteria [25]. Metabolic status was assessed using the Adult Treatment Panel III criteria [26], and having less than two of the following criteria was defined as metabolically healthy: (1) high triglycerides (≥ 1.7 mmol/L), (2) elevated SBP (≥ 130 mmHg) or DBP (≥ 85 mmHg), (3) high FBG (≥ 5.6 mmol/L), and (4) low HDL-C (< 1.04 mmol/L for men and < 1.29 mmol/L for women).

Participants were divided into four metabolic phenotypes according to their combination of BMI and metabolic status: (1) metabolically healthy normal weight (MHNW, BMI between 18.5 and 23.9 kg/m^2^ with zero or one metabolic abnormality), (2) metabolically healthy overweight or obese (MHO, BMI ≥ 24 kg/m^2^ with zero or one metabolic abnormality), (3) metabolically unhealthy normal weight (MUNW, BMI between 18.5 and 23.9 kg/m^2^ with two or more metabolic abnormalities), and (4) metabolically unhealthy overweight or obese (MUO, BMI ≥ 24 kg/m^2^with two or more metabolic abnormalities).

### Ascertainment of NAFLD

Abdominal ultrasonography scanning was performed for all participants after overnight fasting by well-trained staff using Toshiba Nemio 20 sonography machine (Toshiba, Tokyo, Japan) with a 3.5-MHz probe [27]. Based on hepatic ultrasonic examination results, participants were dichotomized into two groups: with or without NAFLD.

### Statistical analyses

The characteristics of participants in different groups were compared using Chi-square tests for categorical variables and one-way analysis of variance/Kruskal–Wallis H test for continuous variables. Logistic regression model was used to estimate odds ratios (ORs) and 95% confidence intervals (CIs) of NAFLD prevalence according to different metabolic phenotypes. All the models were basically adjusted for age and sex and further adjusted for alanine transaminase, total bilirubin, albumin, creatinine, and uric acid. Collinearity was diagnosed for all covariates using variance inflation factor prior to regression analyses. To further explore the role of hyperuricemia in the relationship between metabolically phenotypes and NAFLD, we performed stratified analysis according to uric acid level. Additionally, we further examined the interaction effect by incorporating the two variables (i.e., metabolic phenotypes and hyperuricemia) and their cross-product term in the same model.

In the sensitivity analysis, we repeated the logistic regression models stratified by age and sex. The level of statistical significance was set at a *P* value less than 0.05. All statistical analyses were performed using Stata SE 15.0 for Windows (StataCorp, College Station, Texas).

## Results

### Characteristics of the study population

Among all participants (n = 2959, mean age: 55.02 ± 16.59), 835 (28.2%, mean age: 50.24 ± 16.64) were MHNW, 878 (29.7%, mean age: 52.12 ± 15.70) were MHO, 312 (10.5%, mean age: 65.76 ± 15.70) were MUNW, and 934 (31.6%, mean age: 58.41 ± 15.26) were MUO, respectively. Compared to participants in MHNW, those with MHO were older, males, and had higher BMI, SBP, DBP, triglyceride, HDL-C, LDL-C, creatinine, uric acid, and alanine transaminase, and those with MUNW and MUO were older, males, and had higher BMI, SBP, DBP, total cholesterol, triglyceride, HDL-C, LDL-C, FBG, creatinine, uric acid, and alanine transaminase. There were no significant differences among four groups with respect to total bilirubin and albumin (Table 1). The detailed characteristics results of all participants and characteristics by fatty liver status were shown in Additional file 1: Table S1.

**Table 1:** Characteristic of the study population (n = 2959)

	MHNW (n = 835)	MHO (n = 878)	MUNW (n = 312)	MUO (n = 934)	*P*
Age, years	50.24 ± 16.64	52.12 ± 15.70^a^	65.76 ± 15.70^a,b^	58.41 ± 15.26^a,b,c^	< 0.001
Sex					
Male	432 (51.74)	683 (77.79)	209 (66.99)	764 (81.80)	< 0.001
Female	403 (48.26)	195 (22.21)	103 (33.01)	170 (18.20)	
BMI, kg/m^2^	21.81 ± 1.44	26.46 ± 2.08^a^	22.51 ± 1.21^a,b^	27.39 ± 2.63^a,b,c^	< 0.001
SBP, mmHg	121.55 ± 16.21	129.33 ± 16.92^a^	141.65 ± 16.71^a,b^	141.92 ± 16.19^a,b^	< 0.001
DBP, mmHg	69.95 ± 9.87	75.34 ± 10.46^a^	77.01 ± 11.05^a^	81.68 ± 11.65^a,b,c^	< 0.001
Total cholesterol, mmol/L	5.29 ± 0.96	5.27 ± 0.97	5.49 ± 1.13^a,b^	5.45 ± 1.09^a,b^	< 0.001
Triglyceride, mmol/L	1.16 ± 0.48	1.39 ± 0.61^a^	2.01 ± 1.13^a,b^	2.53 ± 1.77^a,b,c^	< 0.001
HDL-C, mmol/L	1.55 ± 0.32	1.39 ± 0.27^a^	1.28 ± 0.31^a,b^	1.17 ± 0.23^a,b,c^	< 0.001
LDL-C, mmol/L	3.02 ± 0.78	3.20 ± 0.79^a^	3.22 ± 0.87^a^	3.24 ± 0.84^a^	< 0.001
FBG, mmol/L	4.87 ± 0.70	4.97 ± 0.68	6.07 ± 2.25^a,b^	5.96 ± 1.84^a,b^	< 0.001
Creatinine, μmol/L	62.98 ± 17.91	68.52 ± 14.77^a^	67.05 ± 18.56^a^	70.60 ± 29.85^a,b,c^	< 0.001
Uric acid, μmol/L	328.58 ± 82.98	383.65 ± 90.02^a^	358.95 ± 95.66^a,b^	403.16 ± 90.89^a,b,c^	< 0.001
ALT, U/L	16.00 (12.00–22.00)	20.00^a^ (16.00–29.00)	19.00^a,b^ (14.00–25.00)	25.00^a,b,c^ (18.00–37.00)	< 0.001
Total bilirubin, μmol/L	13.00 (10.40–16.20)	13.50 (10.58–16.80)	12.70 (10.30–16.00)	13.20 (10.40–16.90)	0.064
Albumin, g/L	43.79 ± 2.89	43.56 ± 2.47	43.69 ± 3.24	43.77 ± 3.44^a^	0.357

Data are presented as mean ± standard deviations, or number (proportion %)

*MHNW* metabolically healthy normal weight, *MHO* metabolically healthy obesity, *MUNW* metabolically unhealthy normal weight, *MUO* metabolically unhealthy obesity, *BMI* body mass index, *SBP* systolic blood pressure, *DBP* diastolic blood pressure, *HDL-C* high density lipoprotein, *LDL-C* low density lipoprotein, *FBG* fasting blood glucose, *ALT* alanine transaminase

^a^Compared to MHNW group, the difference was statistically significant

^b^Compared to MHO group, the difference was statistically significant

^c^Compared to MUNW group, the difference was statistically significant

### Relationship between metabolic phenotypes and NAFLD

**Fig. 2:**
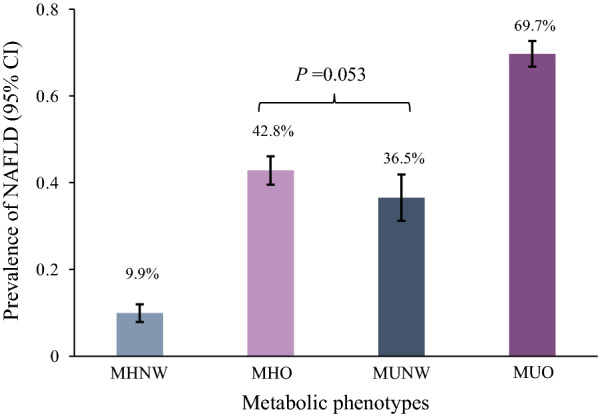
Prevalence of nonalcoholic fatty liver disease by metabolic status

Among all participants, 1224 (41.4%) participants had NAFLD. The prevalence (95% CI) of NAFLD in MHNW, MHO, MUNW, and MUO groups was 9.9% (7.9–12.0%), 42.8% (39.5–46.1%), 36.5% (31.2–41.9%), and 69.7% (66.8–72.6%), respectively (Fig. 2). In the multi-adjusted logistic model, compared to participants with normal BMI, those with overweight (OR 3.06, 95% CI 2.49–3.78) and obesity (OR 7.96, 95% CI 5.92–10.70) were significantly associated with higher NAFLD prevalence. The multi-adjusted ORs (95% CIs) of NAFLD were 1.81 (1.38–2.38) for one metabolic-related factor, and 3.50 (2.62–4.69) for two, 5.71 (4.06–8.04) for three, and 11.77 (6.31–21.94) for four compared to zero metabolic-related factors. There were dose-dependent relationships between higher BMI or the number of metabolic-related factors and higher prevalence of NAFLD (Table 2).

**Table 2:** Odds ratios (ORs) and 95% confidence intervals (CIs) of nonalcoholic fatty liver disease in relation to body mass index (BMI) and metabolic-related factors

	Subjects	Un-adjusted OR (95%CI)	Basic adjusted^a^ OR (95%CI)	Muti-adjusted^b^ OR (95%CI)
BMI				
< 24	1147	Reference	Reference	Reference
24–28	1323	4.62 (3.83–5.57)	4.34 (3.59–5.25)	3.06 (2.49–3.78)
≥ 28	489	16.81 (12.93–21.86)	15.76 (12.06–20.59)	7.96 (5.92–10.70)
* P*_trend_		< 0.001	< 0.001	< 0.001
Metabolic-related factors^c^				
0	721	Reference	Reference	Reference
1	992	2.72 (2.14–3.44)	2.82 (2.21–3.61)	1.81 (1.38–2.38)
2	744	6.13 (4.80–7.84)	6.8 (5.29–8.96)	3.50 (2.62–4.69)
3	410	11.91 (8.92–15.90)	14.36 (10.56–19.52)	5.71 (4.06–8.04)
4	92	22.78 (12.98–39.97)	31.70 (17.75–56.62)	11.77 (6.31–21.94)
* P*_trend_		< 0.001	< 0.001	< 0.001

^a^Adjusted for age, and sex

^b^Adjusted for age, sex, alanine transaminase, total bilirubin, albumin, creatinine, and uric acid, and BMI, and metabolic-related factors if application

^c^Metabolic-related factors refer to four metabolically unhealthy statuses, i.e., high triglycerides (≥ 1.7 mmol/L), elevated SBP (≥ 130 mmHg) or DBP (≥ 85 mmHg), high FBG (≥ 5.6 mmol/L), and low HDL-C (< 1.04 mmol/L for men and < 1.29 mmol/L for women). 0 represent participants who had no metabolically unhealthy status; 1 represent participants who had anyone metabolically unhealthy status; 2 represent participants who had any two metabolically unhealthy status; 3 represent participants who had any three metabolically unhealthy status; 4 represent participants who had four metabolically unhealthy status

In the multi-adjusted logistic model, compared to participants with MHNW, the ORs (95% CIs) of NAFLD were 5.32 (4.01–7.04) for those with MHO, 4.51 (3.17–6.40) for those with MUNW, and 13.68 (10.23–18.30) for those with MUO, respectively (Table 3).

**Table 3:** Odds ratios (ORs) and 95% confidence intervals (CIs) for the association between metabolic status and nonalcoholic fatty liver disease

	Subjects	Un-adjusted OR (95%CI)	Basic adjusted^a^ OR (95%CI)	Muti-adjusted^b^ OR (95%CI)
MHNW	835	Reference	Reference	Reference
MHO	878	6.79 (5.22–8.83)	6.56 (5.02–8.58)	5.32 (4.01–7.04)
MUNW	312	5.22 (3.78–7.21)	6.07 (4.33–8.50)	4.51 (3.17–6.40)
MUO	934	20.84 (15.97–27.20)	2169 (16.44–28.63)	13.68 (10.23–18.30)

^a^Adjusted for age, and sex

^b^Adjusted for age, sex, alanine transaminase, total bilirubin, albumin, creatinine, and uric acid

### Relationship between metabolic phenotypes and NAFLD stratified by uric acid

Considering high uric acid (OR 1.88, 95% CI 1.55–2.28) was independently associated with a higher prevalence of NAFLD, we further analyzed the relationship between metabolic phenotypes and NAFLD by stratification of uric acid levels. In the stratified analysis by uric acid, the prevalence of NAFLD was significantly higher in participants with MHO, MUNW, and MUO in the hyperuricemia group than those in the normal uric acid group (Table 4). There was statistical interaction between metabolic phenotype and uric acid concerning NAFLD (*P* < 0.05).

**Table 4:** Odds ratios (ORs) and 95% confidence intervals (CIs) for the association between metabolic status and nonalcoholic fatty liver disease, stratified by uric acid

	Subjects	Un-adjusted OR (95%CI)	Basic adjusted^a^ OR (95%CI)	Muti-adjusted^b^ OR (95%CI)
Normal serum uric acid				
MHNW	692	Reference	Reference	Reference
MHO	545	5.36 (3.90–7.36)	5.02 (3.64–6.92)	4.55 (3.27–6.35)
MUNW	215	4.89 (3.31–7.21)	5.00 (3.33–7.50)	3.88 (2.55–5.90)
MUO	525	19.33 (14.06–26.58)	18.19 (13.05–25.35)	13.43 (9.54–18.92)
Hyperuricemia				
MHNW	142	Reference	Reference	Reference
MHO	332	7.30 (4.41–12.08)	8.31 (4.95–13.95)	7.82 (4.57–13.41)
MUNW	96	4.62 (2.52–8.46)	6.82 (3.59–12.95)	6.44 (3.33–12.44)
MUO	409	16.85 (10.15–28.00)	21.61 (12.72–36.72)	17.05 (9.82–29.59)

^a^Adjusted for age, and sex

^b^Adjusted for age, sex, alanine transaminase, total bilirubin, albumin, and creatinine

### Supplementary analysis

The results were not materially altered compared to those from the initial analysis when we repeated the following analyses by further performing stratified analysis by sex or age to address possible sex/age differences (Additional file 1: Tables S2 and S3).

## Discussion

In this population-based study of Chinese adults, we found that overweight and obesity remained risk factors for NAFLD independent of metabolically unhealthy phenotypes. Abnormality weight (overweight and obesity) and metabolic disorders posed a joint effect on the risk of NAFLD, and MUO individuals had the highest risk of developing NAFLD. Serum uric acid levels may modify the association between metabolically phenotypes and NAFLD, and the risk effects of MHO, MUNW, and MUO on NAFLD were more evident among individuals with hyperuricemia.

The current study found that the NAFLD prevalence in a physical examination population was 40.5% (46.0% in men and 27.8% in women), and was 9.9%, 42.8%, 36.5%, and 69.7% in participants with MHNW, MHO, MUNW, and MUO, respectively. Chen et al. reported an overall 46.6% prevalence of NAFLD in a community-based population in Taiwan, China [28]. Compared with the study, our research had a low prevalence of NAFLD, and the cause of this difference might be our younger study population (mean age 55.02 years vs. 62.9 years). Zhou et al. using systematic review and meta-analysis methodology summarized the epidemiological features of NAFLD in China and found that the prevalence of NAFLD reached 32.9% in 2018 [29]. Currently, the global prevalence of NAFLD is estimated to be approximately 25%, with a similar prevalence in Asia, where the estimated pooled prevalence is 27.4% [30]. Compared with these results of general population, our study population had a higher prevalence of NAFLD. Furthermore, in the present study, there was no difference in NAFLD prevalence between MUNW and MHO groups, which may be due to age, BMI, and metabolic factors difference between two groups. However, the prevalence of NAFLD in these two groups was still significantly higher than in MHNW groups, suggesting that any abnormality in either BMI or metabolism required attention to the development of NAFLD.

Our findings that MHO was associated with a higher prevalence of NAFLD confirmed several previous reports [3, 12, 29, 31–33]. One cross-sectional study demonstrated that both obesity and metabolically unhealthy status had greater risk of NAFLD after full adjustment for confounders [28]. Likewise, a cohort study that included 77,425 men and women reported that being overweight and obese were significantly associated with over twofold higher risk of developing NAFLD in metabolically healthy men and women[12]. Furthermore, Sailimai et al. conducted a health checkout cohort study that included 31,010 adults, and found that both MHO and MUO were linked with higher risk of NAFLD [34]. Our findings were in line with the abovementioned studies, and the results provided convincing evidence to support that abnormal weight, including overweight and obesity, regardless of metabolic abnormalities, was able to increase NAFLD risk, suggesting that metabolically healthy overweight and obese individuals might still benefit from maintaining a healthy weight to prevent NAFLD.

We found that serum uric acid levels may modify the relationship between metabolic phenotypes and NAFLD, and the risk effects of MHU, MUNW, and MUO on NAFLD were more evident among individuals with hyperuricemia. The association of higher serum uric acid with the risk of prevalent NAFLD had been demonstrated by cross-section studies and prospective cohort analyses [27, 35–37]. A systematic review and meta-analysis included nine observational studies—including four cross-sectional studies, three retrospective reports, and two prospective studies, suggesting that elevated serum uric acid level, independent of conventional NAFLD risk factors, was associated with higher risk of NAFLD [38]. Wei et al. conducted a 4-year prospective cohort study recruiting 2383 health examination population of Chinese adults and found that there was a dose–response relationship between serum uric acid levels and the risk of developing NAFLD, and hyperuricemia may be an independent predictor for NAFLD [27]. Similar to their results, our study confirmed hyperuricemia was related to higher risk of prevalent NAFLD. Furthermore, it was worth noting that our study also provided new evidence that serum uric acid level may modify the association between metabolic phenotypes and NAFLD, and elevated serum uric acid may aggravate the harm of MHU, MUNW, and MUO to NAFLD.

Mechanisms that relate to the interaction between elevated uric acid and metabolically phenotypes associated with NAFLD are still unclear. The potential explanation may be that the coexistence of these two disorders may jointly induce insulin resistance, which was implicated in the pathogenesis of NAFLD [39–41]. Accumulated evidence suggested that metabolic disturbance can bidirectionally induce insulin resistance [42, 43], and metabolically healthy/unhealthy overweight and obesity were closely related to insulin resistance. Still, the association between serum uric acid and insulin resistance has been observed. Some reports suggested that serum uric acid per se might inhibit insulin receptor substrate 1 (IRS1) and Akt insulin signaling and induce insulin resistance [44, 45]. Additionally, hyperuricemia could increase the production of reactive oxygen species (ROS), and both uric acid and ROS could activate the pro-inflammatory factors, which played an important role in the development or worsening of insulin resistance [46–48]. Therefore, the co-occurrence of elevated uric acid and metabolically healthy/unhealthy overweight and obesity may jointly increase the risk of prevalent NAFLD through worsened insulin resistance. Furthermore, another possible mechanism was the additional role of visceral fat in hyperuricemia and NAFLD. Elevated visceral fat has been shown to be associated with the occurrence of NAFLD [49]. This may be due to free fatty acids (FFA) in visceral adipose tissue promoting deposition of hepatic lipids, while visceral adipose tissue and its macrophages also secrete large amounts of pro-inflammatory factors (such as TNF-α and IL-6). The changes in these cytokines caused or aggravated hepatic and peripheral insulin resistance and acted a key role in various stages of liver disease [50]. Moreover, the accumulation of visceral adipose was more likely to cause uric acid metabolic disorders, which by causing hyperinsulinemia to increase renal tubular uric acid reabsorption, or excess FFA through the pentose phosphate pathway to increase purine synthesis [51]. In view of these findings, both visceral adipose itself and hyperuricemia induced by visceral adipose were closely related to the risk of NAFLD.

The strengths of our study included a large sample and the use of standardized diagnoses by the trained sonographer and doctor. Furthermore, our research involved detailed information on demographic and clinical factors, allowing us to adjust for potential confounders. In addition, the participants included in our study focuses on middle aged and older, which is conducive to our research for those individuals at high risk of prevalent NAFLD. To our knowledge, this is the first study to provide the positive messages that serum uric acid levels may modify the association between metabolic phenotypes and NAFLD, thus having important clinical and public health relevance. Our study also had limitations. Firstly, although the study has adjusted for potential sources of bias, there remained unmeasured confounding factors, such as smoking status, physical activity, diet pattern, drug use record, and comorbidities, which may have influenced our risk estimates. Furthermore, information on waist circumference was not available, thus waist circumference was not used in the metabolic phenotypes indicators and could not assess the influence of visceral adiposity on NAFLD. Second, the cross-sectional design of this study did not prove causality, and large population-based prospective cohort studies are needed to confirm our findings. Thirdly, the participants were more likely to have a higher socioeconomic status because most of them were employees of companies and university teachers, limiting the generalizability of the findings to other population groups. Finally, considering the difference between Chinese and other races, thus caution is also needed when generalizing our findings to other populations.

## Conclusion

In summary, our findings suggested that MHO, MUNW, and MUO were associated with a high risk of developing NAFLD. Serum uric acid levels may modify the association between metabolically phenotypes and NAFLD, and the risk effects of MHO, MUNW, and MUO on NAFLD were more evident among individuals with hyperuricemia. Our results highlight individuals with abnormal metabolic phenotypes should pay more attention to the management of serum uric acid levels.

## Supplementary Information

**Additional file 1. Table S1.** Characteristic of study population by fatty liver status (n=2959). **Table S2**. Odds ratios (ORs) and 95% confidence intervals (CIs) for the association between metabolic status and nonalcoholic fatty liver disease, stratified by age. **Table S3**. Odds ratios (ORs) and 95% confidence intervals (CIs) for the association between metabolic status and nonalcoholic fatty liver disease, stratified by sex.

## Data Availability

The original data supporting the study are available from the first author or corresponding author subject to compliance with laws and regulations.

## Abbreviations

BMI: Body mass index
CI: Confidence intervals
DBP: Diastolic blood pressure
FFG: Free fatty acids
FPG: Fasting blood glucose
HDL-C: High-density lipoprotein cholesterol
IRS1: Inhibit insulin receptor substrate 1
LDL-C: Low-density lipoprotein cholesterol
MHNW: Metabolically healthy normal weight
MHO: Metabolically healthy overweight or obese
MUNW: Metabolically unhealthy normal weight
MUO: Metabolically unhealthy overweight or obese
NAFLD: Nonalcoholic fatty liver disease
OR: Odds ratios
ROS: Reactive oxygen species
SBP: Systolic blood pressure
