# Supplementary material for: Hyperuricemia as an effect modifier of the association between metabolic phenotypes and nonalcoholic fatty liver disease in Chinese population
Source: J Transl Med. 2023 Jan 21;21:39. doi: 10.1186/s12967-022-03850-5 (PMC9867866; doi:10.1186/s12967-022-03850-5)
Supplement: Supplementary file 1 — Additional file 1. Table S1. Characteristic of study population by fatty liver status (n=2959). Table S2. Odds ratios (ORs) and 95% confidence intervals (CIs) for the association between metabolic status and nonalcoholic fatty liver disease, stratified by age. Table S3. Odds ratios (ORs) and 95% confidence intervals (CIs) for the association between metabolic status and nonalcoholic fatty liver disease, stratified by sex. [file 12967_2022_3850_MOESM1_ESM.docx]

**Additional file 1**

**Table S1**. Characteristic of study population by fatty liver status (n=2,959).

**Table S2**. Odds ratios (ORs) and 95% confidence intervals (CIs) for the association between metabolic status and nonalcoholic fatty liver disease, stratified by age.

**Table S3**. Odds ratios (ORs) and 95% confidence intervals (CIs) for the association between metabolic status and nonalcoholic fatty liver disease, stratified by sex.

**Table S1**. Characteristic of study population by fatty liver status (n=2,959).

|  | All participants | No-fatty liver  (n=1,735) | Fatty liver  (n=1,224) | *P* |
| --- | --- | --- | --- | --- |
| Age, years | 55.02±16.59 | 54.77±17.61 | 55.36±15.03 | 0.324 |
| Sex |  |  |  |  |
| Male | 2088 (70.56) | 1118 (64.44) | 970 (79.25) | <0.001 |
| Female | 871 (29.44) | 617 (35.56) | 254 (20.75) |  |
| BMI, kg/m^2^ | 25.03±3.19 | 23.76±2.65 | 26.82±3.03 | <0.001 |
| SBP, mmHg | 132.41±18.57 | 129.11±18.79 | 137.08±17.21 | <0.001 |
| DBP, mmHg | 76.00±11.67 | 73.36±11.10 | 79.74±11.45 | <0.001 |
| Total cholesterol, mmol/L | 5.35±1.03 | 5.29±1.02 | 5.45±1.03 | <0.001 |
| Triglyceride, mmol/L | 1.75±1.28 | 1.40±0.90 | 2.25±1.54 | <0.001 |
| HDL-C, mmol/L | 1.36±0.32 | 1.44±0.34 | 1.24±0.24 | <0.001 |
| LDL-C, mmol/L | 3.17±0.82 | 3.08±0.80 | 3.28±0.82 | <0.001 |
| FBG, mmol/L | 5.37±0.82 | 5.18±1.22 | 5.65±1.72 | <0.001 |
| Creatinine, μmol/L | 67.46±21.95 | 66.77±25.19 | 68.43±16.25 | 0.031 |
| Uric acid, μmol/L | 371.68±93.85 | 348.42±87.25 | 404.64±93.03 | <0.001 |
| ALT, U/L | 20.00 (15.00-29.00) | 17.00 (13.00-23.00) | 26.00 (19.00-38.00) | <0.001 |
| Total bilirubin, μmol/L | 13.20 (10.40-16.60) | 13.20 (10.40-16.60) | 13.20 (10.40-16.50) | 0.517 |
| Albumin, g/L | 43.71±3.00 | 43.51±3.02 | 43.98±2.95 | <0.001 |

Data are presented as mean ± standard deviations, or number (proportion %).

Abbreviations: MHNW, Metabolically healthy normal weight; MHO, metabolically healthy obesity; MUNW, metabolically unhealthy normal weight; MUO, metabolically unhealthy obesity; BMI, Body mass index; SBP, Systolic Blood Pressure; DBP, Diastolic Blood Pressure; HDL-C, High Density lipoprotein; LDL-C, Low Density Lipoprotein; FBG, Fasting Blood Glucose; ALT, Alanine transaminase

**Table S2**. Odds ratios (ORs) and 95% confidence intervals (CIs) for the association between metabolic status and nonalcoholic fatty liver disease, stratified by age.

|  | Subjects | Un-adjusted  OR (95%CI) | Basic adjusted ^a^  OR (95%CI) | Muti-adjusted ^b^  OR (95%CI) |
| --- | --- | --- | --- | --- |
| Age <60 |  |  |  |  |
| MHNW | 611 | Reference | Reference | Reference |
| MHO | 636 | 8.44 (6.12-11.65) | 7.23 (5.21-10.03) | 6.13 (4.32-8.68) |
| MUNW | 119 | 8.45 (5.35-13.37) | 8.07 (5.08-12.81) | 6.91 (4.25-11.25) |
| MUO | 525 | 32.32 (22.90-45.62) | 26.83 (18.91-38.08) | 17.44 (9.54-18.92) |
| Age ≥60 |  |  |  |  |
| MHNW | 224 | Reference | Reference | Reference |
| MHO | 242 | 4.04 (2.54-6.42) | 4.34 (2.72-6.95) | 3.89 (2.39-6.32) |
| MUNW | 193 | 2.99 (1.83-4.88) | 3.10 (1.90-5.08) | 2.68 (1.62-4.45) |
| MUO | 409 | 10.71 (6.94-16.52) | 11.51 (7.41-17.88) | 8.95 (5.69-14.08) |

^a^ Adjusted for sex.

^b^ Adjusted for sex, alanine transaminase, total bilirubin, albumin, creatinine, and uric acid.

**Table S3**. Odds ratios (ORs) and 95% confidence intervals (CIs) for the association between metabolic status and nonalcoholic fatty liver disease, stratified by sex.

|  | Subjects | Un-adjusted  OR (95%CI) | Basic adjusted ^a^  OR (95%CI) | Muti-adjusted ^b^  OR (95%CI) |
| --- | --- | --- | --- | --- |
| Male |  |  |  |  |
| MHNW | 432 | Reference | Reference | Reference |
| MHO | 683 | 5.21 (3.82-7.12) | 5.36 (3.91-7.33) | 4.47 (3.21-6.22) |
| MUNW | 209 | 3.26 (2.20-4.83) | 4.34 (2.88-6.54) | 3.43 (2.24-5.27) |
| MUO | 764 | 13.70 (10.02-18.73) | 15.73 (11.41-21.68) | 10.41 (7.43-14.58) |
| Female |  |  |  |  |
| MHNW | 403 | Reference | Reference | Reference |
| MHO | 195 | 8.07 (4.82-13.53) | 7.86 (4.69-13.19) | 6.55 (3.81-11.24) |
| MUNW | 103 | 11.38 (6.40-20.23) | 9.65 (5.30-17.59) | 7.44 (3.97-13.94) |
| MUO | 170 | 45.89 (27.70-78.87) | 39.71 (22.71-69.44) | 23.25 (12.95-41.73) |

^a^ Adjusted for age.

^b^ Adjusted for age, alanine transaminase, total bilirubin, albumin, creatinine, and uric acid.
